# Reworking the Social Determinants of Health: Responding to Material‐Semiotic Indeterminacy in Public Health Interventions

**DOI:** 10.1111/maq.12586

**Published:** 2020-06-27

**Authors:** Emily Yates‐Doerr

**Affiliations:** ^1^ School of Language, Culture, and Society, Oregon State University; ^2^ Department of Anthropology University of Amsterdam

**Keywords:** public health, health intervention, nutrition, inequity, wicked illnesses

## Abstract

Both public health experts and medical anthropologists are concerned with how health is shaped by environmental forces. This creates an important cross‐disciplinary alliance, yet crucial differences in how the two disciplines tend to evaluate health remain. In this article, I compare public health's “social determinants of health” framework with anthropological interest in the sociality of health and illness. I draw on ethnographic fieldwork in Guatemala's highlands, to unpack (1) “the social,” (2) “determinants,” and (3) “of health.” Ultimately, I show how the social determinants framework is deployed in ways that risk undermining its stated health justice goals, and highlight the benefits of an approach that does not know what health is ahead of doing research and which works closely with communities to respond to the effects of its own intervention. The article argues for the need to rework the emphasis on social determinants to make space for health's material‐semiotic indeterminacy

## Wicked Illnesses, Root Problems

In 2005, as I was preparing to move to Guatemala to study the rising diagnosis of obesity in the country, the World Health Organization (WHO) launched a Commission on Social Determinants of Health. The *social determinants* framework, which has since become prominent in global health, seeks to draw attention to *social* conditions of disease.^1^ The framework is central to global conversations about “wicked” illnesses, including obesity and other so‐called non‐communicable diseases that develop slowly, lacking an immediate or easily discernable causal agent. Departing from an approach to improving health that focuses on individual bodies and behaviors, the social determinants framework emphasizes how health outcomes are shaped by the social milieu, including income, educational opportunities, access to housing, and food security. This would seem aligned with anthropological approaches, yet fundamental differences persist.

A common explanation of the social determinants evokes a river. An influential review paper on the topic offers the image of people drinking water contaminated by toxic chemicals originating from a factory upstream (Braveman et al. 2011). The proximate, or downstream, response would be to suggest that individuals buy filters; the upstream response would focus on ending the factory's dumping. The authors note that because there are “multiple intervening and potentially interacting factors” between the dumping and the drinking, it is generally easier to study and address downstream determinants. This, however, contributes to a failure to address “root causes” and also—because wealthier individuals can buy better filters—exacerbates health inequalities (Braveman and Gottlieb 2014).

Many in the field of global health laud the social determinants framework for addressing the fundamental causes of illness in its attention to upstream determinants. Social determinants were founded, in part, to foster a global movement to promote health equity. The very first sentence of a foundational WHO report is that “social justice is a matter of life and death” (CSDH 2008, iii; Marmot et al. 2008). The report explains:

The poor health of the poor, the social gradient in health within countries, and the marked health inequities between countries are caused by the unequal distribution of power, income, goods, and services, globally and nationally, the consequent unfairness in the immediate, visible circumstances of peoples’ lives—their access to health care, schools, and education, their conditions of work and leisure, their homes, communities, towns, or cities—and their chances of leading a flourishing life. This unequal distribution of health‐damaging experiences is not in any sense a “natural” phenomenon but is the result of a toxic combination of poor social policies and programmes, unfair economic arrangements, and bad politics. Together, the structural determinants and conditions of daily life constitute the social determinants of health and are responsible for a major part of health inequities between and within countries. (CSDH 2008, 1)

This explicit concern for social justice has had appeal for social scientists working in health and development fields, many of whom have adopted the social determinants of health framework as their own. Medical anthropology students at a university where I work are required to take a class called Global Health and Care, which they attend with students in the medical school. In the course they read key public health articles on the social determinants of health (e.g., Braveman et al. 2011), and the lectures that surround the readings encourage them to think about how inequalities in health originate in social structures. This is not an apolitical lesson: Locating the cause of poor health in social structures places responsibility in social (and not individual) action, encouraging social (and not individual) transformation.

Despite its stated goals, however, the social determinants framework may uphold and even exacerbate conditions of inequality by prioritizing and targeting a form of health that has been predetermined by distant experts. Its concern for justice and inequality has an inspiring ring; but its reliance on “determinacy” doesn't go far enough in challenging—and changing—the health care systems in which it operates. My concern is that the desire to address the roots of a pre‐given problem imagined to begin at a measurable point and to then advance to a predictable (i.e., determinant) place, sets us on a path toward prescriptive solutions that often do not result in the deep structural transformation they claim to inspire. In contrast, as I illustrate in this article, material‐semiotics offers a different method for responding to and caring for health. It asks how health comes to matter to people in their lives, undertakes interventions in conversation with the people whose lives are impacted by public health's interventions, and insists that external expertise be attuned to existing community‐based expertise, treating both health and health interventions as relational processes.

Material‐semiotics is an approach to knowledge production that considers how meanings and material worlds form together. Donna Haraway, who helped develop material–semiotics, offers this short primer:

It matters what matters we use to think other matters with; it matters what stories we tell to tell other stories with; it matters what knots knot knots, what thoughts think thoughts, what descriptions describe descriptions, what ties tie ties. It matters what stories make worlds, what worlds make stories. (Haraway 2016, 9; see also Haraway 1988; Strathern 1988)

Deploying the double entendre of mattering to mean both giving‐importance‐to and physicality, material‐semiotics emphasizes that ideas and material forms co‐constitute one another. In pithy terms: Concepts matter. Material‐semiotics advances a feminist commitment to addressing the processes by which research questions become askable or erased, as well as the findings that then ensue (see also Asdal 2012; Brives et al. 2016). Instead of seeking universally applicable knowledge, definitions, or answers it is a method of inquiry that makes obvious the absence of generally shared terms, showing how even the lines of inclusion or exclusion drawn around the “we” who is thinking or telling stories are worked out variously. It asks that we attend to material specificities in theories and to how these theories are worked out in people's lives. In this sense, material‐semiotics complements the anthropological method of ethnography by asking for long‐term commitment to people and problems as they change over time, making a case for the importance of learning to respond to different communities of expertise (Yates‐Doerr 2017).

This article proceeds with a material‐semiotic analysis of each of the terms in the social determinant of health framework. I draw on fieldwork in Guatemala's highlands undertaken between 2008 and 2019, in which I have studied how public health workers design and deploy maternal nutrition interventions with the aim of impacting health in a cluster of rural Indigenous Maya‐Mam communities. I analyze this fieldwork to unpack (1) “the social,” (2) “determinants,” and (3) “of health.” Ultimately, I show how the social determinants framework is deployed in ways that risk undermining its stated health justice goals and highlight the benefits of a method of research and analysis that responds to health's *Material‐Semiotic Indeterminacy*.

## “The Social”

“The social” is a good place to start my analysis of the social determinants of health, since this is a seemingly shared concern of the *social* science of anthropology and public health's *social* determinants of health. While the word is obviously used in both fields, it functions quite differently in each, with public health workers frequently operationalizing the social as factor to measure whereas anthropologists treat sociality as fundamental aspect of living based on relations and exchange.

Before I turn to further flesh out the differences in the disciplinary approaches, some historical background on the social is necessary. Drawing on Annemarie Mol's (In press) analysis of the development of Western scientific fields, a brief history of the term might read as follows:

Over the 19^th^ and 20^th^ century, western academic traditions divided the realm of the material from the realm of the social, giving natural scientists jurisdiction over the study of nature while directing social scientists toward the study of society. This was not an even split. Academic institutions treated nature as more real, more fundamental—more true—than society, which was treated as a mess of symbols, meanings, values and viewpoints constructed over it. In the late 20^th^ century, however, social scientists began to turn to study the natural sciences. What they learned was that nature was put into practice in ways that depended on these values and viewpoints. At the same time, natural scientists were finding that the “nature” they studied was thoroughly socialized. The nature/culture split, one of the great dividing lines around which western thought had been organized, began to dissolve.

**Figure 1:**
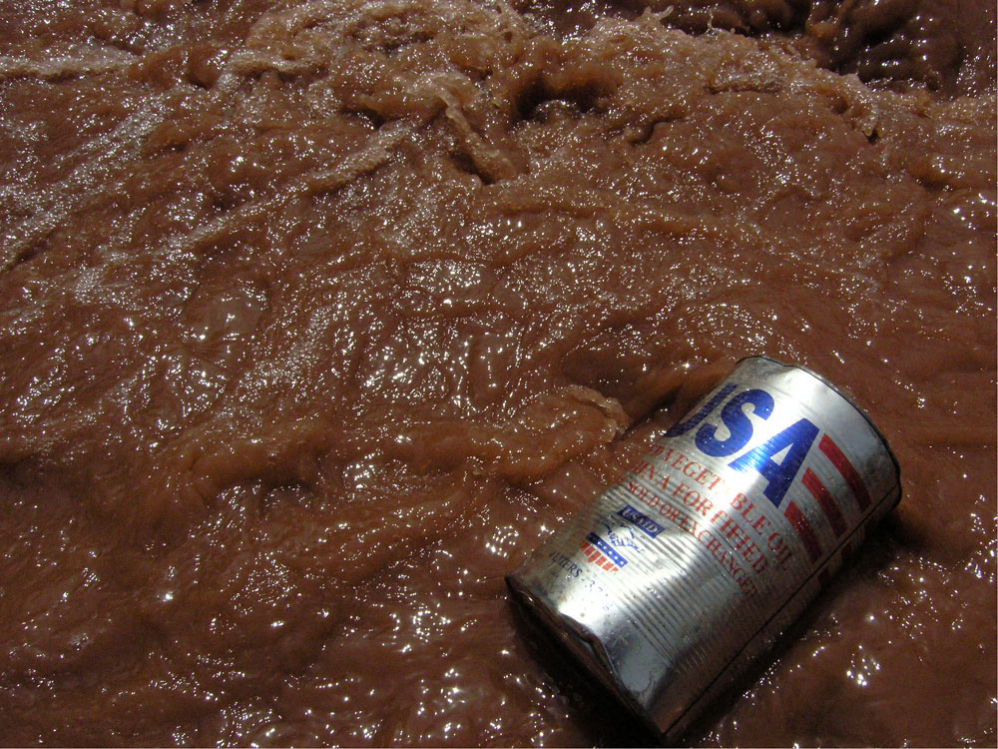
An empty container of oil floats in a river in highland Guatemala. Produced by USAID, this oil is vitamin A‐fortified vegetable oil, “not to be sold or exchanged.” [Photo Credits: the author] [This figure appears in color in the online issue]

Here lies an important point of alliance between anthropology and public health, both of which are interested in the social conditions that give shape to material realities. Consider that the social determinants framework has emerged to counter the focus on the individual's body, with the social seeking to draw attention away from genetic determinants, for example, and toward the environmental in which individuals live. Take the canonical metaphor of the river used by social determinants proponents: The factory and its chemicals, the filter, even the flow of the water are part of its social operations because they have been engineered by humans who have made their design decisions on the basis of social values (Figure 1). The example of toxins flowing downstream is offered to upend the idea that poor health is an outcome of personal knowledge or choice. Even the ability to purchase a filter depends on access to economic resources, which are part of a social system.

Yet even as anthropologists and public health practitioners might, in principle, share a common interest in structural over individual‐level health interventions, there are reasons why anthropologists might not celebrate the placement of the social at the center of a major health and development framework. Adams et al. help explain the difference between the fields, showing how many of the methodologies employed by global health practitioners treat the social as a constant, rather than as “a teeming source of information that may be key to both efficacy and critique of the interventions being attempted” (2019, 1395). Indeed, if we pay attention to how the social is deployed in discussions of social determinants, we see that even as attention toward the social is encouraged, concern for sociality becomes foreclosed. In the practical application of the framework, the social becomes reified as a pre‐established and fixed set of attributes: a measurable piece of a larger, objective, natural world. The relational aspects of the research process diminished, the social becomes treated as a metric to be calculated and known, not inherent to the practices of modelling or apprehending world systems.

For example, the 2008 Commission on the Social Determinants of Health (CSDH) report defines the social as a set of “conditions in which people are born, grow, live, work, and age” (CSDH 2008, 1). These so‐called social conditions are routinely linked to other conditions: “political, economic, *and* social systems”; “physical, mental, *and* social well‐being”; “social *and* emotional learning,” and so on. While the conjunction “and” may seem innocuous enough, as Margaret Lock (2015) notes, this conjunction represents the social as self‐contained: a factor, measurable and distinct from other factors in the equation. It makes it possible for there to be environmental problems that are not always‐already social problems, emotions that are not always‐already social, politics that are not always‐already social—and so on. Treating the social as a metric minimizes the dynamic and unruly forms that life conditions, and the political actions to impact them, will take.

John Law's examination of the scientific survey offers clarity about the difference (2015). He describes one approach to survey taking as follows: Surveys are conducted; statistics are carried out on the sample; findings are scaled up; health warnings are disseminated; a picture of the collective emerges. There is nothing inherently wrong with doing surveys; the problem that Law draws attention to is what happens in stealth. Surveys report on a population with attributes, configuring the social as a collection of individuals, but they tend to do so in a way that substitutes the practice of reporting for the report itself. As a result, the social comes to appear as a preexisting entity to be measured, not as an entity that measurement practices bring about. Meanwhile, anthropological attention to sociality would follow the effects of the survey (e.g., how and by whom it is made, how it works, the kinds of knowledge it makes possible, what it leaves out). In other words, rather than *do* surveys, the material‐semiotic approach would study what the survey *does*, inquiring into the broader effects surveys have on those who make surveys as well as on whom they are carried out.

As a concrete example, the social determinant of health framework often calculates “years of education” to statistically correlate this with health outcomes, whereas attention to sociality might ask for a deep analysis of which, and whose, kinds of education are valued and or ignored, and what the ramifications are of this value system on people's lives (see also Biruk and McKay 2019.). Whereas the public health social determinants framework measures how *social* conditions impact the pre‐given outcome of health, material‐semiotics would instead attend to the sociality of the framework—i.e., the lively ways that life conditions are experienced, stabilized, or transformed.^2^ Instead of taking the meaning of symbols or the measurement of realities as given, it asks how symbols affect the worlds they symbolize, how this feeds back into the symbol, how this then shapes the worlds they symbolize, and so on.

Consider the photograph from Guatemala's highlands I have included here, which shows an oil container floating in a dirty stream showing the letters “USA.” Zoom in on the image, and you will see that the container is of vegetable oil—not gasoline—and I can tell you, by having walked the river's banks during different seasons, that the river's brown color is a result of earth churned up from recent rains—not necessarily undesirable in itself. That this particular oil can's pollution comes from vegetables and not fossil fuels is, however, no cause for relief. As with all USAID goods in Guatemala, the package says, in English, “not to be sold or exchanged,” indicating that the product wasn't meant for the marketplace. But if you spend even a little bit of time in the community you will see exchanges between the U.S. and Guatemalan governments are everywhere, often with devastating consequences on Guatemalan people's lives.

As the river flows out of town it passes the regional cemetery, which is filled with coffins painted with the colors of the U.S. flag—a small indicator of the thousands of people from the region who have recently died trying to cross the national border. If you talk to people about the history of development aid in their region, you will learn that numerous market and military exchanges have structured USAID's presence in their community (Ellis 2019). Continue to get to know people and the material conditions in which they live and labor, and you will learn about U.S. and European pesticide imports that exploded during the green revolution, which today leave seedlings vulnerable and cancer on the rise (Arbona 1998; Dowdall and Klotz 2013; Rahder 2020). Hunger alleviation campaigns marketed and sold these pesticides as a source of salvation during the green revolution, but today, Guatemalans I know widely associate them with land devastation and corporeal damage.

At the time of writing, Francisco Lucas, a journalist from the region where I took the oil canister photograph, is being held under arrest for no obvious reason except that his demands to clean up and protect highland rivers posed challenges to a substantial foreign‐run hydroelectric dam project in the region—and that he is Maya‐Q'anjob'al and a known “water defender” (Ernesto Choc 2019). If you start looking into Lucas's history, you'll learn about how anti‐Indigenous genocide in the 1980s forced survivors from his community into exile in the United States, where they lived as low‐paid agricultural workers and mushroom pickers (O'Connor 2008). Those who have returned to Guatemala face ongoing and often deadly racism, which is especially fierce in this majority‐Indigenous department of Guatemala (Stephen 2019). The brutal U.S.‐aided military general Efraín Ríos Montt, was born and raised here, and the techniques of physical and psychological abuse that he honed during his scorched earth campaign at the height of Guatemala's violence in the 1980s continue to be pervasive. That the established political parties do not value Indigenous life and frequently sanction health projects that have no beneficial health impact is widely recognized, by both academics (Cerón 2019; Velásquez Nimatuj 2019) and highlanders alike. The sociality here at stake is that the very fact of working for better health poses a challenge to powerful economic and state interests who will, in turn, work to make health projects ineffective or to shut these efforts down.

Public health interest in the social determinants of health has arisen because of stated interest in the “unequal distribution of power” (Hunter 2010). The framework was meant to encourage upstream actions, such as cleaning up dirty water at the source—a classic example of the framework's importance and efficacy (Marmot 2017). Yet, to follow this metaphor through, it is often the case that water is not only polluted at an upstream source of entry, but by the lead pipes through which the water moves, the rusty hoses out of which it runs, or the endocrine‐disrupting chemicals leaching from the plastic containers in which it is stored (Liboiron 2018). Sometimes the agencies claiming to fix the pollution may make the problem worse; sometimes entire water systems are toxic.

Instead of delineating the social as a particular bounded aspect of living that will determine downstream health outcomes, I advocate a method that responds to the variability of variables. For the situation described in Guatemala, it would be important to consider how global inequalities are maintained by racist development aid, neoliberal capitalism, and elite‐dominated national politics of the country. While the social determinants of health framework might reduce these concerns to the variables of “political economy,” material semiotics would insist on the importance of asking how politics and economics take shape in specific places. Key to the method of material semiotics is to not impose explanatory models from a distance, but in conversation with the people whose lives models are meant to impact. This method neither attempts to model the social nor to use the social to model health, but enquires into how people value health in their lives and asks what would help create conditions that can make these values matter. This is an approach grounded in lived conditions instead of models, where the socio–material realities in which people live “come first” (Biehl and Petryna 2013).

To further clarify differences in the two approaches, I turn to an analysis of the next term in the framework: *determinants*.

## “Determinants”

*“We are concerned with ‘the causes of the causes’,”* a spokesman for the social determinants explained to me at a workshop to discuss the co‐occurrence of obesity and climate change (see also CSDH 2008:42; Marmot et al. 2012, 1012). Held at the University College London, the workshop sought to bring together interdisciplinary experts to address the conjoined problems of non‐communicable chronic illness, agricultural development, and malnutrition “in all its forms,” to quote a catch phrase that the WHO uses to characterize its work on hunger. The premise of the workshop was that in the case of wicked, synchronistic problems such as obesity and climate change, it was especially important to target “upstream” or “root cause” determinants.

I have learned through interviews with epidemiologists and by attending conferences such as this one, that addressing “the causes of the causes” does several things when it comes to shaping nutrition research and policies. Most obviously, it shifts the conversation away from an individual's food choices and toward structural conditions that influence how bodies develop as they grow. It also directs attention toward intergenerational relations through which metabolic illness emerges instead of toward the individualized patient‐body of biomedicine. A newspaper headline that ran while I was studying obesity in Guatemala reported, “*You are what your grandmother ate*,” encapsulating the message, common among epidemiologists, that obesity develops slowly, with small inputs at one point in fetal development having large repercussions on human and international development years—sometimes even generations—later (Yates‐Doerr 2011; see also Lappé et al. 2019; Valdez 2018).

Again, a shift in focus from individual choice toward long‐term structural forces would seem to be a point of obvious connection between the fields of public health and anthropology, and a reason for anthropologists to embrace the framework of the social determinants of health. And yet, thinking of health in terms of upstream causal factors—even thinking in terms of the causes of the causes—creates distance between the person in question and the malady. This distance, in turn, enables the establishment of health of health and inequity—interventions that frequently further the disparity at hand.

**Figure 2:**
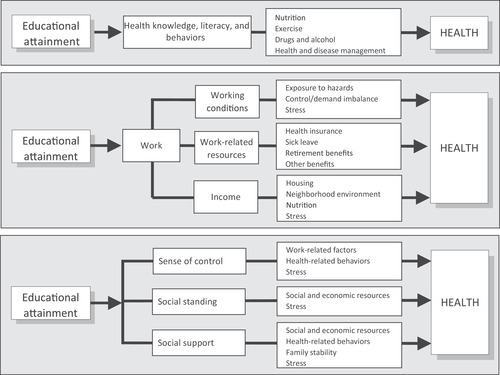
Multiple pathways linking education to health. This graphic depicting how educational attainment determines health was included in the annual review article, “Social Determinants of Health: Coming of Age,” by Braveman, Egereter, and Williams (2011:286).

With *determinacy* as an underlying metaphor, the social determinants literature is full of unidirectional arrows. Take the diagram featured in the review article mentioned above, shown here, which depicts how “educational attainment” determines the outcome health (Figure 2). While the arrows run in one direction, others in public health have suggested that educational attainment is not a cause, but an effect: an outcome of structural inequality. And still others, including myself, have suggested that treating education as a cause of poor health instantiates the troubling message that those individuals diagnosed with weight‐related malnutrition are ignorant, ultimately exacerbating rather than ameliorating structural inequality (Sanabria 2016; Yates‐Doerr 2015).

The concept of determinants is used frequently in mathematics, where determinants are the causal factors of an equation's outcome. Yet, when put into practice, determination frequently works through what Julie Guthman calls “the embedded assumptions of problem closure,” where researchers create models that embed their own values into the models, allowing for causality to be derived from unexamined correlations (2013, 142). Guthman offers the case of epidemiologists who were concerned for *the obesogenic environment*—a term that points to how environmental conditions such as “cheap, fast, nutritionally inferior food and a built environment that discourages physical activity” predispose their inhabitants to obesity (2013, 142). She describes how an unquestioned definition of the problem was used to frame the subsequent study of obesity's causes and consequences in such a way that foreclosed alternative conceptualizations such as environmental toxins, which were not part of the model‐builders’ understanding (Guthman 2013). Researchers, she shows, neglected the possibility that the features of the built environment they studied might be as much of an effect as a cause of the problems they sought to illuminate.

Ashante Reese (2019) and Hanna Garth (2018) extend Guthman's critique of problem closure by pointing to how the field of public health's interest in establishing more grocery stores in places researchers deem to be “food deserts” elides how anti‐black racism frames both the problem of obesity and shapes the trajectories for its solutions. Garth gives the example of anti‐obesity programs in South L.A. that promote organic food markets, while ignoring the stress of police brutality or harmful effects of redlining that would require deep social reckoning and repair to redress. The effect is not only to fail to help, but to further pathologize these communities. The social determinants framework similarly allows certain “acceptable” structural problems to become visible and treatable, while downplaying problems that might more substantively challenge the structures that the framework operates within. For example, the social determinants framework frequently addresses education, school food problems, or nutritional education, while racism, colonialism, or dispossession from land are often considered outside of the purview of social determinants (e.g., Green and Zook 2019).

Material‐semiotics departs from the linear, determinant modeling of the social determinants framework by insisting on the importance of attending to feedback loops, gaps in the model, and even how modeling comes to shape the way that problems are framed and intervened on. It rests on the premise that causes and effects are *indeterminant*, meaning that they cannot be known or defined outside of the systems that bring them into being. Indeterminacy‐based modeling resists the idea of a closed model, asking instead for a form modeling capable of attending to the effects of the model.

The example of height is useful for illustrating the relevance of indeterminacy since this seemingly tangible and fixed measurement becomes obviously indeterminate when taken up in practice. In the months I studied obesity, I learned that health workers in Guatemala placed considerable emphasis on human stature, frequently using height as a proxy indicator for health inequity. Unlike weight, which was known to fluctuate rapidly, height served as a barometer of long‐term hunger. In fact, public health workers directly equated height to the poor health that is caused by chronic malnutrition, and with this, to structural inequality. Whereas epidemiologists have correlated height and ill‐health to make population‐level comparisons, height has come to have clinical implications, with doctors and health extension workers using height to make individual‐level assessments about health. Being short, a doctor explained, could result in decreased cognitive ability and educational performance. The WHO additionally links height to lost productivity at work and excessive weight gain that itself corresponded to an increased incidence of chronic diseases later in life.

When Guatemalan children were young, health workers placed considerable attention on growth, regularly assessing a child's height against WHO growth curves. The logic underlying the practice of growth measurement is that children living in good conditions would grow in a linear and patterned way up until puberty, after which height did not fluctuate much except to decrease slightly and slowly. This logic allowed experts to treat height as a reliable indicator of childhood nutrition: Measuring height would reveal the quality (or inequality, as the case might be) of the social conditions in which an individual was raised.

Yet I saw in my research that even height was unruly, not revealed by the measurement but negotiated through social interactions, capturing something other than what it purported to capture (see also Cabot 2013). When I paid attention to how health workers used height metrics in their practice, I learned that these metrics were often tools that would allow other intimacies to form. For example, nutritionists frequently engaged in the impersonal act of measuring a patient's height because it was a known routine that would ease the patient into the consultation, allowing them to warm up to the more difficult and personal conversations that would unfold when the measurement was over. They actually didn't care, they frequently told me, about the number.

These nutritionists may not have cared about the measurements, however the measurements would continue to exert structural power, as seen in the actions of employers who would privilege taller workers, or people who would, in turn, work to make their bodies taller than they would be otherwise. Consider, for example, the following exchange over reported and measured height between a health worker and the Maya‐Mam woman she is measuring:

Health Worker: You are 147 centimeters tall.
Patient: I've lost five centimeters!
HW: Yes.
P: I thought I was 1 meter and 52 centimeters. That's what my identification card says.
HW: What happens with the identification cards is that one always asks to be taller.
P: A person can shrink ma'am. When someone grows older they get shorter.
HW: I have in my identification card that I'm 155, but I'm not. I'm 154.
P: So the card's a liar then?
HW: Yes.
P: Okay then.

Gideon Lasco (2018) shows how height in the Philippines served the capitalist–colonialist project of U.S. empire. U.S. doctors and scientists who traveled to the Philippines touted height as evidence of U.S. superiority and used height minimums to exclude Filipinos from civil, military, professional, and leisure activities. Lasco details the extreme and often painful activities that some Filipinos began, in response, to undertake to become tall. These included: ingesting commercial “height building” supplements; avoiding carrying heavy objects; using height‐enhancing sleep regimens; undertaking physical bone‐stretching routines that might include walking on tiptoes or wearing high‐heeled shoes; and male circumcision (associated with growth) (Lasco 2017). Being tall had material consequences; Lasco presents a chart correlating height and salary to argue that “height was an undeniable determinant of employment chances” (Lasco 2018). While public health workers treat height as an outcome of upstream determinants, height also becomes the determinant.

In Guatemala, the maneuver from “shortness is unhealthy” to “short people are themselves undesirable” is often so slight as to be imperceptible. I was told—and have observed myself—that domestic servants (frequently referred to through the derogatory term “*muchachas”*) were typically very short because the stigma facing very short women made other forms of employment impossible. It is not coincidental that Indigenous Guatemalans are, on average, shorter than non‐Indigenous Guatemalans, with a majority of Maya people in Guatemala widely referred to as “stunted” (at least two standard deviations below WHO's reported averages of height‐for‐age). In Guatemala, short stature is not only evidence of inequality to be overcome, but was widely associated with poor fitness and used to further discriminate against Indigenous people, serving as a means of holding inequality in place. While the social determinants framework takes health to be determined by the social environment, this is only one of the truths of the story. Also true is that what is taken to be health will determine which—and how—factors are measured.

During the agri–health conference mentioned at the start of this section, it became clear to me that even as health and policy experts emphasized getting to the “the causes of the causes,” assembling causational models whose arrows pointed outward in one direction, cyclical reasoning was common. Several of the speakers contributed to the following year's Global Nutrition Report, which listed “food supply, clean water and sanitation, education, and health care” as underlying drivers of good nutrition (Haddad 2014). That the report also pointed to nutrition as a key determinant of these drivers—particularly the drivers of education and health care—illustrates how muddled causal pathways can be.

Instead of an approach that promotes linear determinacy, we need an approach for addressing wicked problems that allows for looping and feedback and responds to how problems are shaped by their attempted solutions. After all, determinants will have indeterminate effects—i.e., effects that can't be known in absolute terms, but that form and change as they are put into practice in the everyday. Material‐semiotic *indeterminacy* takes the very practice of model‐making as a necessary piece of the model. As applied to the situation of nutritional health in Guatemala, it would turn away from the language of determinants to instead ask about the social and historical conditions of how people live, what they want, and how these conditions and desires change over time. It doesn't act from afar in a linear direction, but acts by engaging, listening to, and adjusting itself in response to how it is taken up by the people whose lives it seeks to impact.

## “of Health”

At the heart of the social determinants framework lies the idea that there is an outcome—heath—which is determined by social conditions. No matter that it is extremely difficult to collect good data on statistics of morbidity or mortality (e.g., Gerrets 2015), at least in theoretical terms, the framework treats health as a metric. This contrasts with a material‐semiotic approach to health, in which health is persistently relational, such that one cannot *be* healthy (as a definable status) but that one *does* health in practice. Much has been written about the dissonance between what health practitioners treat and what people suffer from (e.g. Adams 2016; Napier et al. 2017; Yates‐Doerr and Carney 2016) but another example may still be helpful.

As I carried out fieldwork in Guatemala in 2008–09, I spent several months working with public health providers who delivered a World Food Program product called *Vitacereal* to rural Mam‐speaking Guatemalan communities deemed too poor to adequately feed themselves. The powders were bolstered by the message that early childhood nutrition was the highest priority social determinant of health (Marmot et al. 2012; WHO 2012). They arrived with the promise that intervening into the root cause determinant of fetal malnutrition would have a cascading effect, bettering all aspects on health later in life.

Each bag contained 33 105‐calorie portions (for children) or 10 380 calorie portions (for pregnant and nursing women). The bags were delivered with a cooking lesson, showing women how to add boiling purified water to dissolve the powdered formula, which educators explained held concentrated proteins, fats, and carbohydrates, as well as vitamins and minerals that would, if consumed correctly, make them healthy. After distributing the powders, the health workers collected anthropometric measurements of height and weight, which they would later transfer into a public health database. Epidemiologists would draw on this data to monitor regional trends in rates of chronic malnutrition, which USAID (2014) reports to be among the gravest in the world.

**Figure 3:**
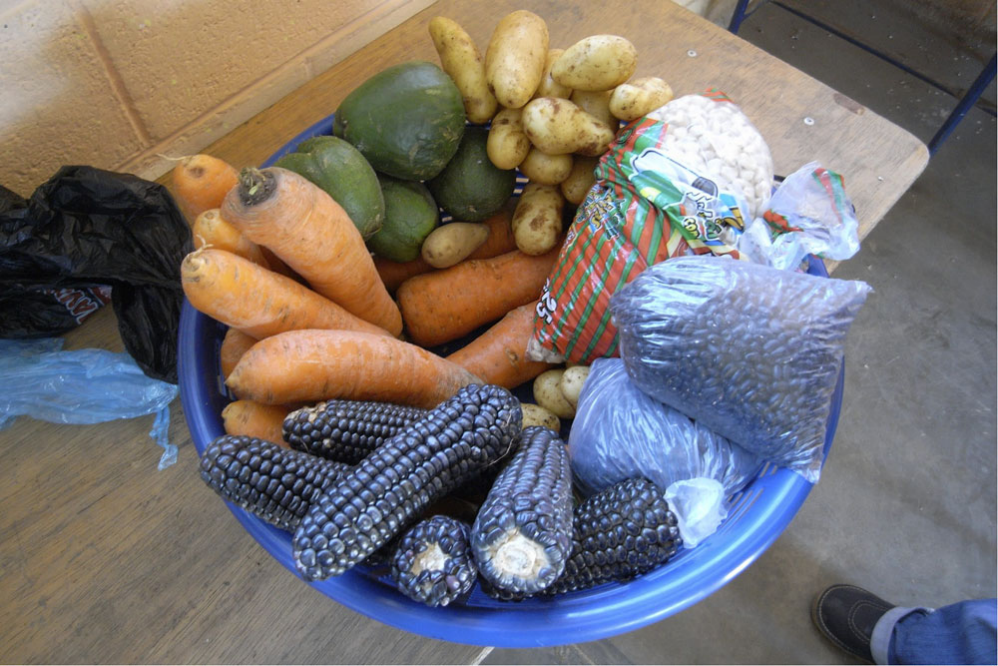
Guatemalan community members collected this gift for public health experts. [This figure appears in color in the online issue]

The week before Christmas, I spent the day with a group of Mam‐speaking women in one of the communities served by the nutrition fortification program, almost all of whom were women since the community's men had been pulled away in search of employment elsewhere in Guatemala and the United States. That day, the group was busy planning a gift for the health workers and I joined along as well. We went door‐to‐door collecting food—whatever the household could spare. We didn't stay too long, but long enough to drink a cup or two of the hot and sweetened instant Nescafé that was typical. Within a few hours of making rounds through the community we had assembled the gift (Figure 3). This was a holiday present, not intended for even reciprocation. Still, the disparity of this exchange has lingered with me: The health workers delivered packages of nutrients; the villagers returned this with corn, avocados, squash, and carrots.

I wanted to ask the women in the community about the significance of the gift—what it meant to them—but stopped before I did. They surely could have responded with reasons why they had exchanged food for nutrient powers, inverting institutional hierarchies with their basket of beautiful produce. And yet asking so directly did not feel right. The question presupposed that their gift could fit into rational, causal orders of exchange. To demand a causal explanation on such a layered and multifaceted action felt wrong, even colonialist in how it forced the expectations of my languages upon other people's lives (Ngũgĩ 1986). So I kept my question to myself, trying to be content with a different kind of explanation: This was what was done.

*Not asking* about health (in Spanish the words for health are *sano* or *salud*—both of which evoke the field and techniques of medicine) when I spent time with people opened up a register of “health”‐related values that we might associate with well‐being, but which were not obviously medical (see also Hardin 2018).^3^ For example, when a small cut on the foot of an elderly diabetic woman refused to heal, became infected, and caused her death, the woman's pregnant daughter, Estella, talked to me shortly after the funeral about how her own son had become lost from her within the U.S.’s immigration system. Over the course of the day I spent with her, she never once mentioned her mother's lack of access to diabetes medication or nutritious food. Health concerns were certainly present in her life; her mother's death had caused intense distress and suffering within her family. Yet there was no category of individual well‐being separated in any meaningful way from economics or migration. People left for the United States because someone in their family had grown ill; they grew ill because someone in their family had left.

Every household in this community had children, aunts or uncles, a parent—sometimes even both parents—who lived without secure documentation in the United States. Many were struggling through systems of indentured servitude, surviving (or not) while trapped in debt to employers, smugglers, or predatory bail bond associations. Employment opportunities in Guatemala were scarce or nonexistent, schools were severely under‐resourced, politicians were in the process of successfully shutting down the anti‐corruption bureau that had recently revealed widespread political malfeasance, and Indigenous community leaders across Guatemala were threatened or assassinated with little recourse. The need for the kind of deep structural reform advocated by the social determinants framework was obvious to everyone with whom I spoke.^4^

What actually happened, however, was that public health programs arrived at the community with packages of vitamin and protein supplements, promising that these would improve the health of pregnant women and their children. People managing their delivery said (with varying levels of conviction) that the nutrient packets were contributing directly to the social determinants agenda. After all, changing physiological pathways of early life growth and development was meant to have impressive “downstream” effects, impacting everything from cognitive processes to chronic health. The desire to impact root cause of early life nutrition was so strong that I frequently heard public health workers say some variation of, “once you've missed this critical window of development, it's too late.” Pregnant women mattered because they were “upstream,” but middle‐aged women? Or women past reproductive years who might be sick or suffering? Or men? One health worker responded to these questions by telling me: “Focusing downstream is not a good application of our limited resources.” He later emphasized the point by adding “We might as well throw money away.”

A framework for social justice action that locates health within the person, and particularly the pregnant person, risks overlooking the degree to which Estella's health is wrapped up in the lives of her mother and her son. From Estella's standpoint, it would have made no sense to overlook her mother because she was downstream. The model might treat pregnant bodies as determinants of future health, discounting the health of mothers and sons. But this logic ignores how Estella lives within social structures, such that the care for elderly people and young boys is essential not only because of its impact on her body but because they are vital members of social systems.

Natali Valdez has shown how “future‐oriented health logics” that drive prenatal health research wind up harming real, living pregnant women in the name of caring for their hypothetical progeny (Valdez Forthcoming; see also Colom 2015). Similarly, I am describing a situation in which caring for pregnancy as an investment in future health creates a system that fails to care for those around the pregnancy—and thus the pregnancy as well. Valdez encourages a platform for justice‐based intervention that focuses on “racist environments” and not individual bodies. As illustrated here, part of the racist environment that must be considered is the environment that produces the Indigenous pregnant body as the primary site on which intervention is thought to be possible.

The social determinants framework says we should look at causes of causes when seeking to improve a person's health. But treating health as if it is located within a person ignores how relations move in waves and circles, foreclosing the many kinds of health that are salient in people's lives. Health workers, arriving with the gift of supplements, placed their focus on what Estella was eating during pregnancy, shifting attention away from the deep and harmful histories of racism and settler‐supremacy that shape affliction in the Americas, her community, her life, and the broader landscape of health intervention. Their ultimate end‐goal of individual health undermines the very focus on health systems that has made the social determinants framework attractive for so many.

## Material Semiotic Indeterminacy

Anthropologists have been drawn to the social determinants of health framework for good reasons, including its stated interests in challenging systems of inequality and transforming existing networks of power. However, the sections above offer reasons why anthropologists might hesitate before wholeheartedly adopting the framework as their own.

In the section elaborating the social, I critiqued how models that treat the social as a bounded and measurable unit elide the power of sociality, in which units come undone—only to be powerfully reassembled in different ways. I unpacked the varied symbolism in a photograph of an oil canister floating in a highland Guatemala stream to point to how stark material inequalities and racist histories continue to structure life in Guatemala and beyond. The framework's stated prioritization of social surroundings instead of individual choice is laudable—but a lesson of material‐semiotics is that it is necessary to attend to ideas as they are put into practice, following how they actually unfold in people's lives.

In the section focused on determinants, I highlighted the often circular or muddled directionality of cause and effect in the modeling of social determinants. For example, nutrition is often treated as a key determinant: something that produces good health. But it is also taken as an effect, with bodies that are in good health being more adept at metabolic regulation. Likewise, height measurements may reveal something about the quality of the social environment in which one grows, serving as an indicator of upstream determinants; but height can also be used as marker of stigma or discrimination, itself becoming the cause of the cause of social inequity. Turning away from linear determinacy, I argued for the importance of methods capable of responding to the indeterminacy of modeling, in which models come to shape the outcomes they portend to reflect.

In the section of health, I took issue with how a framework that seems to value systems and structures might ultimately re‐center the health of the biological individual. I expressed concern with how the persistence of biological health as the end goal of the model allows for deep structures of violence and racism to continue to be ignored. I offered the example of how the focus on upstream determinants becomes mobilized to target fetal health, in the process discounting the importance of the lives of those who fall further downstream. My argument was that a framework that prioritizes the efficiency of targeting upstream determinants such as reproductive health might not, actually, be doing the social justice work that it claims to do.

A possible response to my critique of the framework might be to change the determinant targeted. Instead of improving conventional social determinants of nutrition or education, the focus could be placed on further upstream determinants like racism or gender‐based violence. This would certainly be an important start, but my concern is not only that the wrong determinant is targeted. My concern is also that there is harm in a system unresponsive to how health may not just be determined by social factors but may, conceptually and materially, be social. Put differently, there are significant limitations to targeting deep structural factors such as racism and settler‐supremacy to produce the outcome of better health, if the desired outcome of health is defined and evaluated through harmful structures.

The social determinants of health framework makes models out of social conditions with the stated goal of addressing health equity. As a research technique, material‐semiotics offers a different approach. Instead of calculating the effects of linear determinants, it cultivates the sensibility of iterative relationality and response‐abilities that are grounded in particular places. Law notes that with “this sensibility comes a wariness of the large‐scale claims common in social theory” (2009, 142). Because it situates knowledge in specific places and specific (albeit anonymized) people, it departs from grand‐narrative explanations. Its knowledge is powerful *because* it is specific, not because it claims to be broad, representative research.

I have not outlined material‐semiotics to encourage a revival of amoral cultural relativism, in which there are no languages for talking about health as a good and sickness as suffering. My goal is rather to insist on the methodological necessity of creating interventions that account for, and are responsive to, their own effects. Sara Ahmed's discussion of “non‐performativity” helps to clarify the stakes (2012). Ahmed describes situations where the apparent performance of social justice—she looks specifically at academic programs ostensibly designed to promote diversity—helps undermine the ends these programs claim to be working toward. As she describes it, the appearance of performance holds entrenched and violent systems in place.

In the examples I've offered, projects targeting social determinants and aiming to deeply transform social systems frequently come to reinforce instead of transform existing social structures. Prenatal nutrition, said to strengthen long‐term community health, further burdens some of Guatemala's most precarious communities. Efforts to combat chronic malnutrition reinforce stigma against Indigenous people. Talk of structural transformation has women worried about their diets, opening markets into which the processed or diet food industry will, with the frequent help of public health officials, sell its future‐building technologies and supplements. “Fixing” holds in place the problem it promises to solve (see also Grant 2017; Hardon et al. 2019; Li 2007).

By asking what actually happens to people as ideas move into practices and back, material‐semiotics requires that statements about social justice be linked to their real‐world effects. Tracing the varied everyday life effects of modeling—as it is implemented and over time—prevents us from rallying around concepts or programs that sound good but fail to substantively impact people's lives in ways that they desire for themselves. After all, as Ahmed shows us (2012), even calls for anti‐racism can be used to prevent racist systems from transforming. Following the varied ways that concepts do—or do not—matter in people's lives can help build programs that do not just sound good, but bring about the deep transformations that are promised.

Having written about differences between public health and anthropology's orientations to description and intervention, let me end on a point of convergence. Just as I have critiqued a blanket embrace of the social determinants, neither would I advocate its wholesale dismissal. Instead, following the method of material‐semiotics, I would want to ask how, for whom, and where is the social determinants of health working well? In the case of the maternal nutrition programs I described in Guatemala, reference to social determinants laid the groundwork for health projects that undermined both health and social values. But perhaps elsewhere the social determinants framework is being leveraged to bring about structural transformations that people who stand to benefit find to be useful and good. I would welcome the inspiration of learning where and how the framework might effectively bring about change that people need and desire, and what makes it successful in these cases. From there we might ask how to bring these lessons elsewhere—not scaling them up but attuning them to other socio‐material conditions at hand.

## Notes

1
http://www.who.int/social_determinants/sdh_definition/en/

2 Note that sociality, as I'm using the term, includes non‐human actors in its framework of sociality; to be social is not to be human but to be contingent on relations.

3 Note, however, that the Spanish for well‐being, bien estar, was commonly associated with the nutrient supplement, Bienestarina™ and was, therefore, already biomedicalized.

4
https://www.who.int/social_determinants/en/
